# The *In Vitro* Antiplasmodial Activities of Aqueous Extracts of Selected Ghanaian Herbal Plants

**DOI:** 10.1155/2020/5041919

**Published:** 2020-05-20

**Authors:** Elizabeth Cudjoe, Dickson Donu, Ruth E. Okonu, Jones A. Amponsah, Linda E. Amoah

**Affiliations:** ^1^Noguchi Memorial Institute for Medical Research, University of Ghana, Legon, Ghana; ^2^Department of Biochemistry, Cellular and Molecular Biology, University of Ghana, Legon, Ghana

## Abstract

**Background:**

*The asexual and sexual stages (gametocytes) of Plasmodium falciparum parasites* are known to respond differently to antimalarial drugs. Herbal products with extended treatment regimens and inadequate dosing information are widely used to treat malaria in Ghana. This study set out to determine the *in vitro* activity of selected herbal extracts on the development of asexual and sexual stage malaria parasites.

**Methods:**

The 72-hour SYBR Green 1-based *in vitro* drug assay was used to determine the asexual parasite growth inhibitory effects exhibited by aqueous extracts of *Alchornea cordifolia*, *Polyalthia longifolia*, *Moringa oleifera*, and *Mangifera indica* on the NF54, CamWT_C580Y, and IPC 4912 strains of *Plasmodium falciparum*. The effects of exposure of asexual and early-stage NF54 gametocytes to varying concentrations of the aqueous herbal extracts were assessed by microscopy after 7 days of continuous culturing in the presence of the herbal extract. Qualitative and quantitative phytochemical screening were also performed on the herbal extracts.

**Results:**

In the SYBR Green 1 assay, aqueous extracts of *Alchornea cordifolia* exhibited moderate (IC_50_ of 5.8, 17.4, and 15.8 *μ*g/ml) and *Mangifera indica* exhibited low (IC_50_ of 65.4, 96.7, and 81.7 *μ*g/ml) activities against the NF54, Cam WT_C580Y, and IPC 4912 parasites, respectively, whilst *Polyalthia longifolia* and *Moringa oleifera* were inactive. Long-term treatment of NF54 parasites with 1 mg/ml of *Polyalthia longifolia* produced the highest densities of gametocytes and the least (56%) inhibition of asexual parasites on Day 7. Long-term treatment of NF54 parasites with 10 *μ*g/ml *Alchornea cordifolia* resulted in complete parasite (asexual and gametocyte) clearance on Day 7. *Conclusions. Alchornea cordifolia* exhibited a ‘moderate' activity against the three parasites tested in the 72-hour SYBR Green 1 assay and also effectively cleared both asexual parasites and gametocytes. Long-term treatment of malaria parasites with herbal extracts mimics a treatment regimen and should be used to determine the antimalarial properties of herbal extracts.

## 1. Introduction

The recent reports of artemisinin-resistant and artemisinin-tolerant *Plasmodium falciparum* (*P*. *falciparum*) parasites call for more innovative methods to control and eventually eliminate malaria [1]. Gametocytes, the sexual transmissible forms of the malaria parasite, have been suggested to be produced at each round of asexual replication in the host [2]. Gametocytes develop through five developmental stages: early-stage (stages I, II, and III) also referred to as young gametocytes and late-stage (stages IV and V)/mature gametocytes. Gametocyte development is known to last 10 days in vivo [3]; however, *in vitro*, early-stage gametocytes can be distinguished from asexual parasites after 7 days of continuous culturing [4].

The first-line antimalarial treatment for uncomplicated *Plasmodium falciparum* malaria worldwide comprises the artemisinins, which are a fast-acting class of antimalarials [5] combined with a partner antimalarial drug such as lumefantrine and referred to as artemisinin combination therapy (ACT) [6]. Therapeutic doses of antimalarial drugs are formulated to ensure complete clearance of all infecting malaria parasites; however, drug-resistant parasites or drug-tolerant parasites have evolved that thrive under drug pressure [7]. *Plasmodium falciparum* parasites resistant to the artemisinin class of antimalarial drugs were initially reported in Cambodia [8] and have also been identified in Africa [9]. These artemisinin-resistant parasites are known to produce more gametocytes *in vivo* [10] and consequently enhance malaria transmission.

Consumption of herbal remedies containing extracts of one or more herbs such as *Alchornea cordifolia* (*A*. *cordifolia*), *Polyalthia longifolia* (*P*. *longifolia*), *Moringa oleifera* (*M*. *oleifera*), and *Mangifera indica* (*M*. *indica*) for the treatment of malaria is high in Ghana [4, 11]. In Ghana, the course of treatment for herbal antimalarial products range between 1 and 2 weeks [4]. Exposure of *P*. *falciparum* parasites to suboptimal drug concentrations as well as long-treatment regimens with antimalarial drugs can result in the parasites developing drug resistance [12, 13].

Many studies have determined the antimalarial activity of herbal extracts against the asexual disease-causing parasite using the standard 72-hour drug assay [14–18], and their activity was classified as ‘good' (IC_50_ < 10 *μ*g/ml), ‘moderate' (IC_50_ 10 *μ*g/ml to 50 *μ*g/ml), ‘low' (IC_50_ 50 *μ*g/ml to 100 *μ*g/ml), and ‘inactive' (IC_50_ > 100 *μ*g/ml) based on the concentration of the product that causes a 50% reduction in parasite prevalence (IC_50_) values [19]. The effects of a longer treatment regimen on both the asexual parasite and the gametocyte have more frequently been left undetermined. In this study, the short- and long-term effects of aqueous extracts of four selected herbal plants collected from the Western Region of Ghana on malaria parasite growth in vitro were determined using artemisinin-resistant IPC_4912, the artemisinin sensitive CamWT_C580Y and the chloroquine-sensitive NF54 *P*. *falciparum* parasites.

## 2. Materials and Methods

### 2.1. Identification and Processing of Herbal Plants

Fresh leaves of *Alchornea cordifolia* (*A*. *cordifolia*), *Polyalthia longifolia* (*P*. *longifolia*), *Moringa oleifera* (*M*. *oleifera*), and *Mangifera indica* (*M*. *indica*) were obtained from the Western Region of Ghana and sent to a botanist at the University of Ghana herbarium as well as a Research Officer (Crop Scientist) at the Centre for Plant Medicine Research (CPMR) herbarium, Mampong, Ghana, for identification although no voucher specimens were kept. The leaves were air dried and ground in a blender into a rough powder. The entire amounts of powdered *A*. *cordifolia* (21.5 g), *M*. *indica* (32.0 g), *P*. *longifolia* (17.1 g), and *M*. *oleifera* (6.7 g) leaves were individually boiled at 100°C in 475 ml of H_2_O for one hour. The solutions were then left for 18 hours at room temperature after which they were filtered three times using a Whatman™ 54 filter paper and subsequently freeze dried (lyophilized) using a Labconco™ Freeze Dryer. Stock concentrations (50 mg/ml) of each herbal product were prepared by reconstituting 250 mg of each freeze-dried product into 5 ml of sterile water. The stock solutions were filter sterilized through a 0.2 *μ*m Acodisc™ filter and stored at -20°C for future use.

### 2.2. Culturing of Plasmodium Parasites

Asexual cultures of NF54 (MRA-1000: chloroquine sensitive), CamWT_C580Y (MRA-1250: artemisinin sensitive), and IPC 4912 (MRA-1241: artemisinin resistant) were maintained *in vitro* using a modified method of Zirihi et al. [17] and similar to Amoah et al. [4]. Briefly, the parasites were individually cultured at 4% hematocrit (O+ red blood cells (RBCs)) in complete parasite media (CPM: RPMI 1640 supplemented with HEPES, L-glutamine, NaHCO_3_, glucose, gentamycin, and AlbuMAX II) in a T75 culture flask. The cultures were maintained in an incubator set at 37°C with daily medium change with CPM and exchange of gas (92.5% nitrogen, 5.5% carbon dioxide, and 2% oxygen).

Synchronized ring-stage parasites were obtained by treating a culture containing more than 5% ring-stage parasites with a solution of 5% sorbitol. Two days (48 hours) after synchronization, the cultures, which were predominantly ring-stage parasites were plated at 2% for the SYBR Green 1 assay or 1% parasitaemia for the long-term parasite exposure assay.

### 2.3. 72-Hour SYBR Green I Asexual Parasite Drug Assay

A protocol similar to that described by Quashie et al. [20] and Smilkstein et al. [21] with some revisions was used to determine the inhibitory effects of the aqueous extracts on the different *P*. *falciparum* parasites. Briefly, a 96-well tissue culture plate was filled with five replicates of 50 *μ*l of 20 mg/ml of two herbal extracts serially diluted 10-fold for 6 additional concentrations until 0.02 *μ*g/ml. The first 2 of the quintuplicate wells were supplemented with 50 *μ*l of uninfected RBC (RBCs) set at 2% hematocrit in CPM; the last 3 wells were supplemented with 50 *μ*l of infected RBC (iRBCs) set at 2% parasitemia and 2% hematocrit in CPM. The last set of duplicate wells were filled with 50 *μ*l of infected RBC (iRBCs) set at 2% hematocrit and 2% parasitaemia (ring-stage parasites) in CPM and supplemented with 50 *μ*l of artesunate (20 *μ*g/*μ*l) serially diluted 10-fold for 6 concentrations with CPM until 0.2 ng/ml (schematic of plate set up in Supplementary file 1). The plate was then placed into a Modular incubating chamber and gassed for 6 minutes and then incubated for 72 hours. The plate was then wrapped in aluminum foil and stored at -80°C overnight, after which the plate was thawed at room temperature. Two technical replicate plates were set up for each herbal extract. To each culture-containing well, 100 *μ*l of buffered SYBR Green (2x SYBR Green 1 dye in 20 mM Tris-HCl, pH 7.5 supplemented with 5 mM EDTA, 0.08% Triton X-100, and 0.008% saponin in PBS) was added and mixed up and down. The plate was wrapped again in aluminum foil and stored in an incubator at 37°C for 1 hour. Fluorescence was then read on a microplate reader at 490 nm excitation and 530 nm emission.

### 2.4. Long-Term Parasite Exposure Assay

This assay was replicated from Amoah et al. [4] with some minor modifications. Briefly, each stock herbal product was diluted in CPM to obtain a working solution of 200 *μ*g/ml, 20 *μ*g/ml, and 2 *μ*g/ml. Untreated CPM (no added supplement) was used as a negative control, and 5 ng/ml artesunate was used as a positive control. One hundred microliters of the working concentrations of each herbal product and the control were added to individual wells in a 24 well plate. One hundred microliters of parasite culture at 1% parasitemia and 4% hematocrit was added to the medium-filled well of the plate and placed in a Modular® incubating chamber. The chamber was gassed for 6 minutes with mixed gas (92.5% nitrogen, 5.5% carbon dioxide, and 2% oxygen) and placed in an incubator set at 37°C. The media on the cultures in the plate were changed daily with the initial starting medium (CPM supplemented with 100 *μ*g/ml, 10 *μ*g/ml, and 1 *μ*g/ml of herbal extract) throughout the assay. Thin-film smears were prepared alongside the daily medium change every day for 7 days. The smears were fixed in absolute methanol and stained with 10% Giemsa for 15 minutes. Daily smears were processed and observed under a compound light microscope using a 100x oil immersion objective lens; however, only Day 7 smears were counted against 1000 RBCs for asexual parasites and 5000 RBCs for gametocytes. Each assay was conducted in duplicate and repeated at least twice.

### 2.5. Qualitative Phytochemical Analysis

Phytochemical screening of the individual aqueous herbal extracts was performed using previously published protocols [22–28]. The Frothing test for saponins [29]; Fehling's test for reducing sugars [29]; the Ferric chloride test for phenolic compounds, polyuronides, and cyanogenic glycosides; Mayer's test for alkaloids; Shinoda's test for flavonoids; and the Salkoski test for triterpenes and phytosterols were performed [29]. The presence of anthracenosides was also determined using a protocol described in Benmehdi et al.'s study [25]. All assays were performed with little to no modification to the published protocols and were set up in triplicate and repeated at least twice.

### 2.6. Quantitative Phytochemical Analysis

The total phenolic content of aqueous extracts of *A*. *cordifolia* and *M*. *indica* was determined using the Folin-Ciocalteau method [30–32]. Total flavonoid content was determined using a procedure similar to Madaan et al.'s [32], and the total saponin content was determined using a method similar to that reported by Karimi et al. [33].

All assays were done in triplicate and repeated at least twice.

### 2.7. Statistical Analysis

For the SYBR Green 1 drug assays, the data obtained from the herbal extract-treated uninfected RBC was used as the background and subtracted from the corresponding infected RBC dataset. Data was transformed, normalized, and subjected to a nonfit linear regression test (log(inhibitor) vs. normalized response test) to enable estimation of the 50% inhibitory concentrations (IC_50_) for each herbal product.

The 50% inhibitory concentration (IC_50_) represents the amount of product required to kill 50% of the total parasite content of a sample.

Differences in NF54 parasite response to the 7-day treatment with the herbal products were estimated by two-way ANOVA using GraphPad Prism® 5.0 software package (GraphPad Software, San Diego, CA, USA). The asexual parasitemia and gametocytaemia (early-stage gametocyte) of the continuous culture assay were obtained after counting 5000 RBC per thin smear.

The % inhibition of the asexual parasite growth on Day 7 was calculated in Excel using the formula: (parasitemia in untreated culture–parasitemia in treated culture)/parasitemia in untreated culture × 100].

The influence of 7 days of exposure to the herbal product on gametocyte development was calculated based on the formula: (gametocytaemia in treated cultures/gametocytaemia in untreated culture × 100).

*P* values for statistical significance were set at 0.05 unless otherwise stated.

## 3. Results

### 3.1. Herbal Extract Preparation for Drug Assays

The yield of freeze-dried products from the ground leaves ranged from 9% in *M*. *indica* to 15% in *M*. *oleifera* (Table 1).

### 3.2. Asexual Parasite Growth Inhibition

The 50% inhibitory concentrations (IC_50_) estimated for *A*. *cordifolia* and *M*. *indica* against NF54 were 5.81 *μ*g/ml and 65.36 *μ*g/ml, respectively, and were much lower than those for *M*. *oleifera* and *P*. *longifolia*, which were both over 100 *μ*g/ml (Table 2, Supplementary file 2). *Alchornea cordifolia* and *M*. *indica* were subsequently tested against artemisinin-sensitive (CamWT_C580Y) and artemisinin-resistant parasite (IPC 4912) isolates using the same concentration range, and both extracts *Alchornia cordifolia* and *M*. *indica* exhibited ‘moderate' and ‘low' IC_50_ values for both the artemisinin-resistant and artemisinin-sensitive parasite isolates (Table 2 and Supplementary file 3). *Moringa oleifera* and *P*. *longifolia*, whose antimalarial activity against the NF54 parasite were in the ‘inactive' category, were not further evaluated for activity against the artemisinin-sensitive and artemisinin-resistant parasites.

### 3.3. Long-Term Treatment of Asexual Parasites (AS) with the Herbal Extracts

**Figure 1:**
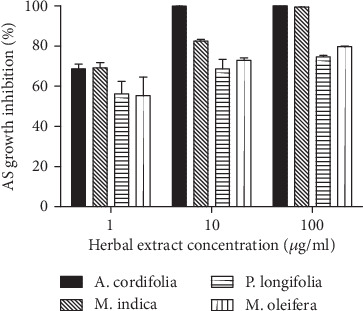
Herbal extract-induced asexual parasite (AS) growth inhibition. A graphical representation of the difference in the number of asexual parasites (AS; rings, trophozoites, and schizonts) identified in each thin smear expressed as a percent of the parasites identified in the control untreated culture (% inhibition). A total number of 5000 RBCs were counted in each thin smear. The data represents the mean (SEM) of the observed growth inhibition.

Cultures of *P*. *falciparum* NF54 parasites treated with 1, 10, or 100 *μ*g/ml of all the four herbal extracts exhibited a significant reduction (*P* < 0.0001, Tukey's post hoc test, Supplementary file 4) in parasite density after 7 days of continuous culturing in treated media compared to parasite density in the untreated media. At 1 *μ*g/ml, the four herbal extract exhibited similar (*P* > 0.05, Tukey's post hoc test) activity on the asexual parasite. Cultures treated with 10 *μ*g/ml of *A*. *cordifolia* exhibited almost 100% parasite inhibition, which was significantly different from the inhibition exhibited by the other three extracts (*P* < 0.05, Tukey's post hoc test). Cultures treated with 100 *μ*g/ml of *M*. *indica*, resulted in 100% parasite inhibition but the cultures treated with 100 *μ*g/ml of *P*. *longifolia* and *M*. *oleifera* reached a maximum of 75 and 80%, respectively (Figure 1). Inhibition was 100% in the positive control cultures, where no parasite (asexual or gametocyte) survived after 7 days of continuous treatment in media supplemented with 5 ng/ml of artesunate.

### 3.4. Gametocyte Development under Constant Treatment with the Herbal Extracts

**Figure 2:**
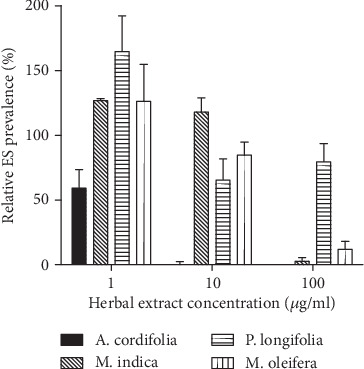
Relative prevalence of early-stage (ES) gametocytes on 7 days. A graphical representation of the total number of early-stage (ES) gametocytes (stage II) identified in each culture after 7 days of continuous exposure to herbal extracts. A total number of 5000 RBCs were counted in each thin smear, and the number of gametocytes counted was expressed as a percent of the number of gametocytes identified in the control untreated culture. The data represents the mean (SEM) number of gametocytes observed. No gametocytes were observed in the artesunate-treated cultures on Day 7.

Seven days of continuous culturing of NF54 parasites *in vitro* with daily medium change without RBC supplementation led to differential gametocyte densities in the treated and untreated cultures (Figure 2). At 1 *μ*g/ml, *A*. *cordifolia* was the only product that exhibited a gametocytocidal activity; the three other products all enhanced gametocyte production. At 10 *μ*g/ml, *A*. *cordifolia* exhibited 99% gametocyte inhibition, which was significantly higher (*P* < 0.0001, Tukey's post hoc test; Supplementary file 4) than the inhibition exhibited by similar concentrations of the other three extracts. *Mangifera indica* produced significantly higher numbers of gametocytes than the untreated cultures even at 10 *μ*g/ml (*P* < 0.05, Tukey's post hoc test). There was a general dose-response effect, with an increase in extract concentration yielding higher gametocytocidal activity and all extracts excluding *P*. *longifolia* exhibiting almost 100% gametocytocidal activity at 100 *μ*g/ml. No significant reduction in the prevalence of gametocytes was observed between cultures treated with 10 *μ*g/ml and 100 *μ*g/ml of *P*. *longifolia*.

### 3.5. Phytochemical Analysis

Qualitative and quantitative phytochemical analyses were conducted on *A*. *cordifolia* and *M*. *indica*, which both exhibited activity against the asexual parasite. Aqueous extracts of both contained reducing sugars, phenolic compounds, and flavonoids. *M*. *indica* contained saponins, which were absent in *A*. *cordifolia* (Table 3). *A*. *cordifolia* had a higher content of both total phenolics and total flavonoids than *M*. *indica* (Table 4).

## 4. Discussion

*Alchornea cordifolia*, *M*. *indica*, *P*. *longifolia*, and *M*. *oleifera* are components of some well-known herbal antimalarial products sold in the Ashanti Region of Ghana [34] although not prominent in herbal antimalarial products sold in the Tema metropolis of the Greater Accra Region of Ghana (Supplementary file 5). A number of studies have characterized the antimalarial properties of some herbal extracts; however, only a few studies have reported the effects that long-term administration of varying concentrations of herbal products have on the malaria parasite [4]. This study determined the IC_50_ values as well as evaluated the long-term effects of exposing malaria parasites to varying doses of the selected herbal extracts on artemisinin-sensitive and artemisinin-resistant malaria in order to determine whether prolonged herbal extract could enhance gametocyte production as well as access their effectiveness against artemisinin-resistant parasites.

In this study, *M*. *oleifera* was found to be inactive against the asexual NF54 *P*. *falciparum* parasite. This is similar to previous findings where methanolic extracts of *M*. *oleifera* exhibited low and no activity against the poW and Dd2 *P*. *falciparum* strains, respectively [35]. The inactivity of *P*. *longifolia* against the NF54 parasite is contrary to a recent report where an aqueous *P*. *longifolia* leaf extract exhibited a moderate activity against the 3D7 parasite strain [36] but supports previous studies where *P*. *longifolia* exhibited activities close to the ‘inactive' range (between 92.6 and 100 *μ*g/ml) against the K1 *P*. *falciparum* parasite strain [37]. However, total methanolic extract of *P*. *longifera* was found to exhibit a ‘moderate' activity (IC_50_ 22.04 *μ*g/ml) against the K1 strain [38].

The 50% effective concentration (EC_50_) of an extract of *A*. *cordifolia* fruit against the 3D7 *P*. *falciparum* parasite strain using the parasite lactate dehydrogenase assay was found to be 4.9 *μ*g/ml [39] which is similar to 5.81 *μ*g/ml of the NF54 strain that was obtained in this study (Table 2, Supplementary file 2). Another study in Ghana estimated the IC_50_ for a 50% ethanolic extract of *A*. *cordifolia* leaves against the 3D7 parasite to be 14 *μ*g/ml using a similar SYBR Green 1 assay [40]. The main difference in IC_50_ values could be attributed to the solvent [41], as the active agent in *A*. *cordifolia* is ellagic acid [42], which is more soluble in water than ethanol. The aqueous extract of *A*. *cordifolia* exhibited a ‘moderate' activity against the artemisinin-resistant parasite (Table 2, Supplementary file 3), which is an excellent indication that it has a potent antimalarial activity that could be used effectively to treat artemisinin-resistant parasites. The aqueous extract of *M*. *indica* exhibited a ‘low' activity against the artemisinin-resistant parasite (Table 2, Supplementary file 2), which suggests that it would not be a good candidate to effectively treat an infection that comprises of artemisinin-resistant parasites. Both *A*. *cordifolia* and *M*. *indica* however exhibited a similar activity against both the artemisinin-sensitive and artemisinin-resistant parasite isolates, suggesting that their possible modes of action against the malaria parasite are different from the mode of action of artesunate, which exhibited a 10-fold difference in 50% inhibitory concentration against the artermisinin-sensitive and artemisinin-resistant parasites (Table 2).

After 7 days of continuous culture of parasites in the presence of herbal extracts, none of the concentrations of *P*. *longifolia* or *M*. *oleifera* tested were able to clear 100% of the infecting parasites (Figure 1). Combined with the fact they exhibited an activity against the malaria parasite in the ‘inactive' range suggest that aqueous extracts of these two herbs do not work independently as effective antimalarial agents.

The increase in gametocyte counts (enhanced gametocyte production) in all cultures treated with 1 *μ*g/ml of the herbal extracts (Figure 2) could be due to the fact that suboptimal drug treatment has been suggested to indirectly increase gametocyte prevalence [12]. *Polyalthia longifolia* exhibited the least gametocytocidal activity at all the tested concentrations and did not exhibit any dose response, suggesting that treatment of malaria with *P*. *longifolia* at concentrations of 100 *μ*g/ml and below may sustain the transmission of malaria transmission due to the persistence of gametocytes after treatment. *Alchornea cordifolia* exhibited the highest gametocydal activity (Figure 2), even at low concentrations suggesting its potential use as a gametocytocydal agent.

*A*. *cordifolia* and *M*. *indica*, which exhibited ‘good' and ‘low' antimalarial activities, respectively, according to the classification by Batista et al. [19], were selected for qualitative phytochemical screening followed by a quantitative phytochemical screening of positive compounds identified in the qualitative screen (Tables 3 and 4). Although some previous studies have reported the phytochemical composition of these two plants, it was important to repeat the analysis on the extracts used in this study because different species of the same plant [43], as well as certain growth conditions, have been noted to influence the phytochemical content of medicinal plants. Phytochemical analysis of herbal extracts obtained from plants grown in different geographical settings reveals vast differences in phytochemical content [44], with soil from the rainforest suggested to be capable of increasing the phenol content of some plants [45].

Flavonoids and phenolic compounds in herbal plants have been associated with antimalarial activity [46] and could be the reason for the much higher activity exhibited by *A*. *cordifolia*, which contained much higher levels of phenolic and flavonoid content relative to *M*. *indica* (Table 4). The phenolic content of *A*. *cordifolia* has been suggested to be mainly composed of ellagic acid, a water-soluble polyphenol that has been suggested to be responsible for the antiplasmodial activity of *A*. *cordifolia* [47].

This study did not use the Ring Survival Assay to assess or validate the artemisinin sensitivities of the different parasites used in this study. Further studies are needed to determine whether *A*. *cordifolia* and *M*. *indica* extracts exhibit a gametocytocidal activity on mature gametocytes. The antimalarial activities of component fractions of *A*. *cordifolia* and *M*. *indica* also need to be investigated.

## 5. Conclusion

*Alchornea cordifolia* exhibited a ‘moderate' activity against the three parasites tested in the 72-hour SYBR Green 1 assay and also effectively cleared both asexual parasites and gametocytes. Long-term treatment of malaria parasites with herbal extracts mimics a treatment regimen and should be used to determine the antimalarial properties of herbal extracts.

## Figures and Tables

**Table 1:** Yield of lyophilized aqueous herbal extract.

Plant	Dried ground leaves (g)	Lyophilized extract (g)	Yield (%)
*A*. *cordifolia*	21.5	2.3	10.7
*M*. *indica*	32.0	2.9	8.9
*P*. *longifolia*	17.1	1.7	9.7
*M*. *oleifera*	6.7	1.0	14.9

The amount of lyophilized extract obtained from the dried leaves expressed as %.

**Table 2:** Activity (IC_50_) of herbal extracts on asexual *P*. *falciparum* parasites.

	NF54	CamWT_C580Y	IPC 4912
Artesunate (ng/ml)	0.499 ± 0.09	0.48 ± 0.18	4.034 ± 1.16
*A*. *cordifolia* (*μ*g/ml)	5.81 ± 1.34	17.42 ± 1.49	15.83 ± 1.39
*M*. *indica* (*μ*g/ml)	65.36 ± 1.20	96.96 ± 1.64	81.68 ± 1.31
*P*. *longifolia* (*μ*g/ml)	161.80 ± 0.97	ND	ND
*M*. *oleifera* (*μ*g/ml)	176.5 ± 0790	ND	ND

ND: not determined; IC_50_: concentration at which 50% of the parasites are killed. The data is presented as the mean ± the standard error of the mean. NF54: chloroquine-sensitive parasite; CamWT_C580Y: artemisinin-sensitive parasite; IPC 4912: artemisinin-resistant parasite.

**Table 3:** Qualitative phytochemical analysis of *A*. *cordifolia* and *M*. *indica.*

Phytochemical	*A*. *cordifolia*	*M*. *indica*
Saponins	-	+
Reducing sugars	+	+
Phenolic compounds	+	+
Cyanogenic glycosides	-	-
Alkaloids	-	-
Anthracenosides	-	-
Triterpenes	-	-
Phytosterols	-	-
Flavonoids	+	+

+ indicates present; - indicates absent.

**Table 4:** Quantitative phytochemical analysis of *A*. *cordifolia* and *M*. *indica.*

	*A. cordifolia* (aq.)	*M*. *indica* (aq.)
Total phenolics (mg GAE/g extract)	91.5 ± 4.0	25.8 ± 1.0
Total flavonoids (mg RE/g extract)	0.876 ± 0.002	0.600 ± 0.005
Total saponin content (mg DE/g extract)	ND	1.801 ± 0.03

ND: not detected; GAE: gallic acid equivalent; RE: rutin equivalent; DE: diosgenin equivalents.

## Data Availability

The data used to support the findings of this study are included within the article.

## Supplementary Materials

Supplementary Materials Supplementary file 1: illustration of SYBR Green 1 assay plate set up. Supplementary file 2: graphical representation of NF54 asexual parasite growth inhibition. Supplementary file 3: graphical representation of IPC 4912 and CamWT_C580Y asexual parasite growth inhibition. Supplementary file 4: statistical analysis of the D7 treated samples. Supplementary file 5: frequency of use of herbal extracts in 19 licensed herbal products.
