# Supplementary material for: The In Vitro Antiplasmodial Activities of Aqueous Extracts of Selected Ghanaian Herbal Plants
Source: J Parasitol Res. 2020 May 20;2020:5041919. doi: 10.1155/2020/5041919 (PMC7256685; doi:10.1155/2020/5041919)
Supplement: Supplementary Materials — Supplementary file 1: illustration of SYBR Green 1 assay plate set up. Supplementary file 2: graphical representation of NF54 asexual parasite growth inhibition. Supplementary file 3: graphical representation of IPC 4912 and CamWT_C580Y asexual parasite growth inhibition. Supplementary file 4: statistical analysis of the D7 treated samples. Supplementary file 5: frequency of use of herbal extracts in 19 licensed herbal products. [file 5041919.f1.docx]

Supplementary file 1. Sybrgreen 1 assay plate layout

Lanes 1-2: 100 μl of HE 1, an equal volume of uninfected RBCs would be added

Lanes 3-5: 100 μl of HE 1, an equal volume of infected RBCs would be added

Lanes 6-7: 100 μl of HE 2, an equal volume of uninfected RBCs would be added

Lanes 8-10: 100 μl of HE 2, an equal volume of infected RBCs would be added

Lanes 11-12: 100 μl of artesunate, an equal volume of infected RBCs would be added

Supplementary file 2. Normalized growth inhibition curves for NF54 treated parasites

Supplementary file 3. Normalized growth inhibition curves for CamWT_580Y and IPC 4912 parasite isolates

Supplementary file 4. Statistical analysis performed for the long term assay

Supplementary file 5. Frequency of use of herbal extracts in 19 licensed herbal products

| Herbal plant | Number of products | |  |
| --- | --- | --- | --- |
| *Azadirachta indica* | 6 | |  |
| *Cryptolepis sanguinolenta* | 5 | |  |
| *Paullinia pinnata* | 3 | |  |
| *Khaya senegalensis* | 3 | |  |
| *Nauclea latifolia* | 3 | |  |
| *Phyllanthus fraternus* | 2 | |  |
| *Xylopia aethiopica* | 2 | |  |
| *Pilostigma* | 2 | |  |
| *Carapa procera* | 2 | |  |
| *Rauwolfia vomitoria* | 2 | |  |
| *Cola gigantea* | 2 | |  |
| *Solanum torvum* | 2 | |  |
| *Spathodea campanulata* | 2 | |  |
| *Bombax buonopozense* | 2 | |  |
| *Vernonia amygdalina* | 2 | |  |
| *Anthocleista nobilis* | 1 | |  |
| *Vifex grandifolia* | 1 | |  |
| *Citrus aurantifolia* | 1 | |  |
| *Psidium guajava* | 1 | |  |
| *Bidens pilosa* | 1 | |  |
| *Veronica conserta* | 1 | |  |
| *Crossopteryx febrifuga* | 1 | |  |
| *Acacia nilotica* | 1 | |  |
| *Gongronema latifolium* | 1 | |  |
| *Terminalla ivorensis* | 1 | |  |
| *Alstonia boonei* | 1 | |  |
| *Monodora myristica* | 1 | |  |
| *Xylopia aethiopica* | 1 | |  |
| *Swietenia macrophylla* | 1 | |  |
| *Albizia ferruginea* | 1 | |  |
| *Aloe schweinfurthii* | 1 | |  |
| *Cassia siamea* | 1 | |  |
| *Ocimum viride* | 1 | |  |
| *Pycnanthus angolensis* | 1 | |  |
| *Azadirachta indica + Cryptolepis sanguinolenta* | | 2 | |
| *Khaya senegalensis + Pilostigma* | 2 | |  |
| *Carapa porcera + Cryptolepis sanguinolenta* | 2 | |  |

Some herbal extracts were found in combinations and the prevalence of these combinations were recoded in addition to the prevalence of the individual herbal extracts. None of the 19 products on sale in Tema, Greater Accra Region of Ghana contained any of the four herbal plants (*A. cordifolia,* *M. indica, P. longifolia and M. oleifera)*
